# A complete response to S-1 plus cis-diamminedichloroplatinum in advanced-stage esophageal and gastric adenocarcinoma: a case report

**DOI:** 10.1186/1477-7819-10-133

**Published:** 2012-07-03

**Authors:** Yoritaka Matsuno, Mitsugu Kochi, Masashi Fujii, Noriaki Kanamori, Teruo Kaiga, Yoshiaki Mihara, Tomoya Funada, Teruyuki Miyazaki, Tadatoshi Takayama

**Affiliations:** 1 Department of Digestive Surgery, Nihon University School of Medicine, Tokyo, Japan

**Keywords:** Chemotherapy, Complete response, Esophageal cancer, Gastric cancer

## Abstract

**Background:**

Complete remission from advanced-stage synchronous double primary (SDP) esophageal and gastric adenocarcinoma by chemotherapy alone is rare. We report a case of advanced-stage SDP esophageal and gastric adenocarcinoma in which a complete response to treatment was obtained with S-1 and cis-diamminedichloroplatinum (CDDP).

**Case presentation:**

The patient was a 74-year-old man referred to our hospital complaining of dysphagia. Gastrointestinal endoscopy was performed and advanced-stage SDP esophageal and gastric adenocarcinoma diagnosed. Computed tomography revealed multiple regional lymph node metastases in the mediastinum. Neoadjuvant chemotherapy with S-1 and CDDP for advanced esophageal and gastric cancer was planned. An endoscopy following two courses of chemotherapy revealed that the esophageal cancer had been replaced with a normal mucosal lesion and the gastric tumor with a scar lesion; the results of biopsies of both were negative for cancer. Computed tomography revealed that the multiple lymph node metastases had disappeared. We diagnosed a complete response to S-1 and CDDP in advanced-stage SDP esophageal and gastric cancer. The patient is still alive with no signs of recurrence 22 months after the disappearance of the original tumor and metastatic lesions without surgical treatment.

**Conclusion:**

These results suggest that complete remission from advanced-stage esophageal and gastric cancer can be obtained with chemotherapy with S-1 plus CDDP.

## Background

The standard treatment for advanced-stage esophageal cancer is esophagectomy. Despite advances in surgical technique, the prognosis of advanced-stage esophageal cancer is still poor [1]. Additionally, some studies have reported that double primary cancer in patients with esophageal cancer has a worse prognosis than a single malignancy [2,3] and that synchronous double primary (SDP) cancer has a worse prognosis than metachronous cancer [4,5]. Several prospective trials have demonstrated that neoadjuvant chemotherapy in conjunction with surgical intervention confers a survival benefit for locally advanced esophageal cancer [6,7]. Recent chemotherapeutic regimens including S-1 have produced good clinical responses and survival benefits in patients with gastric cancer in Japan [8]. Tumor response to an S-1 regimen in patients with advanced esophageal adenocarcinoma, however, remains to be elucidated. Complete remission of advanced-stage SDP esophageal and gastric adenocarcinoma to chemotherapy is rare. One such case is reported here.

## Case presentation

**Figure 1:**
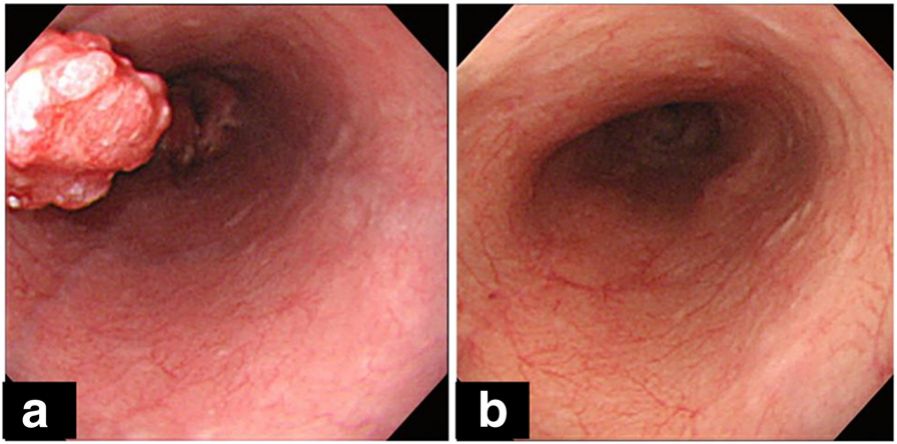
**Upper-gastrointestinal endoscopy before and after treatment of mid-esophageal tumor upper gastric tumor. (a)** In January 2009, initial upper-gastrointestinal endoscopy disclosed mid-esophageal type I tumor measuring 2.5 cm × 2.5 cm. **(b)** In April 2009, follow up after first course of chemotherapy with S-1 and cis-diamminedichloroplatinum, upper-gastrointestinal endoscopy revealed scar lesion instead of tumor; no further lesions were identified.

**Figure 2:**
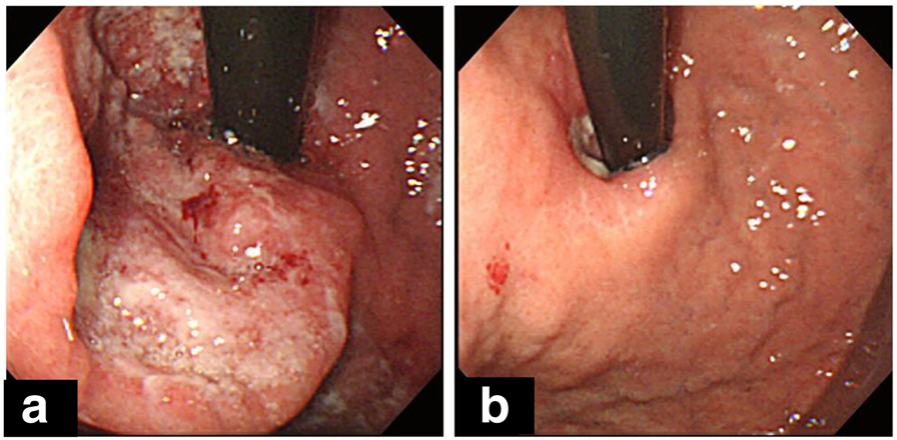
**Upper-gastrointestinal endoscopy before and after treatment of upper gastric tumor. (a)** In January 2009, initial upper-gastrointestinal endoscopy disclosed Borrmann type II tumor measuring 5.0 cm × 4.0 cm in upper gastric tumor. **(b)** In April 2009, follow up after first course of chemotherapy with S-1 and cis-diamminedichloroplatinum, upper-gastrointestinal endoscopy revealed scar lesion instead of tumor; no further lesions were identified.

**Figure 3:**
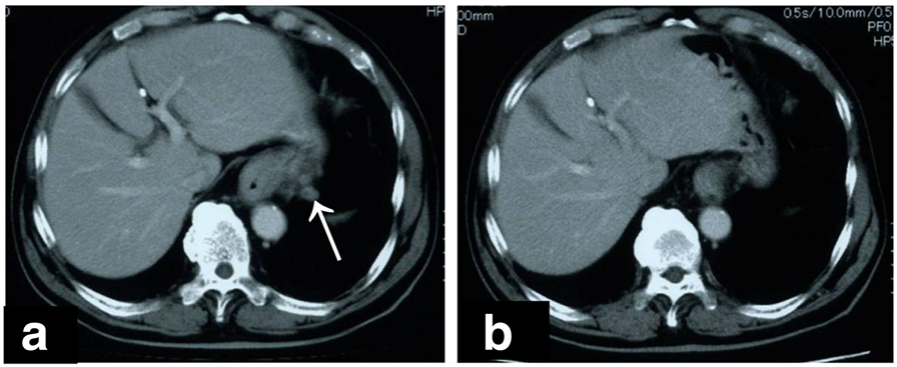
**Computed tomography images before and after treatment.** Initial computed tomography revealed multiple lymph node metastases around esophagus. **(b)** Follow-up computed tomography revealed that multiple lymph node metastases around esophagus had completely disappeared.

The patient was a 74-year-old man who had previously been treated for diabetes, meningitis, hypertension and hyperglycemia by his family doctor. In October 2009, the patient was referred to the Department of Digestive Surgery, Nihon University School of Medicine Itabashi Hospital, complaining of dysphagia. An upper-gastrointestinal endoscopy revealed a mid-esophageal type I tumor measuring 2.5 cm × 2.5 cm (Figure 1a) and an upper-gastric type III tumor measuring 5.0 cm × 4.0 cm (Figure 2a). Biopsy specimens revealed that both tumors were moderately differentiated adenocarcinomas. We diagnosed advanced-stage SDP esophageal and gastric adenocarcinoma. Computed tomography (CT) revealed multiple regional lymph node metastases in the mediastinum (Figure 3a). Neoadjuvant chemotherapy with S-1 (Taiho Pharmaceutical, Tokyo, Japan) and cis-diamminedichloroplatinum (CDDP) was carried out for advanced esophageal and gastric adenocarcinoma. S-1 has recently produced good clinical responses and survival benefits in Japanese patients with gastric and colorectal adenocarcinoma, and hypopharyngeal and laryngeal squamous cell carcinoma. In this case, S-1 was administered orally at a dose of 80 mg/m^2^ per day for 21 consecutive days followed by a 14-day drug-free interval. Infusional CDDP was administered at a dose of 60 mg/m^2^ for 90 min on day 8. The patient developed only grade 1 gastrointestinal dysfunction in all the course of this chemotherapy. An endoscopy performed after two courses of chemotherapy revealed that the esophageal cancer had been replaced with normal mucosa and the gastric cancer with a scar lesion (Figures 1b, 2b); the results of the biopsies of both were negative for cancer lesions. Additionally, CT revealed that the multiple lymph node metastases had disappeared (Figure 3b). We diagnosed complete response of advanced-stage SDP esophageal and gastric adenocarcinoma to S-1 and CDDP as a neoadjuvant chemotherapy, obviating the need for surgical treatment. Over the next 4 months, periodic follow-up was performed to determine whether any further esophageal or gastric lesions had formed. After 4 courses of the S-1 and CDDP chemotherapy regimen, the patient remains on outpatient 8 cycles adjuvant chemotherapy with S-1 only at a dose of 80 mg/m^2^ per day for 28 consecutive days followed by a 14-day drug-free interval without surgical treatment. A periodically performed upper-gastrointestinal endoscopy performed in July 2011 revealed no new tumor lesions and CT has revealed no lymph node metastasis. As of September 2011, the patient is alive with no signs of recurrence at 22 months after the disappearance of the original tumors.

## Discussion

Several retrospective studies have reported that the complete response rate of advanced-stage esophageal cancer to chemotherapy is between 2.0% and 5.6% [9-12]. In contrast, the complete response of advanced-stage gastric cancer to chemotherapy has been reported to be between 0% and 0.7% [13,14]. A complete response of SDP esophageal and gastric adenocarcinoma to chemotherapy is rare. Several retrospective studies have reported a complete response rate of 17% to 36% to chemoradiotherapy for advanced esophageal cancer [6,7,15]. However, radiotherapy is not standard therapy for gastric cancers because there are few cancer of the esophagogastric junction in Japan. Our results suggest that chemotherapy with S-1 and CDDP is an effective treatment for advanced-stage esophageal adenocarcinoma. Recently, the effect of docetaxel and CDDP with docetaxel, cisplatin, 5-fluorourcil (DCF) in the treatment of gastroesophageal cancer was reported. The overall confirmed response rate was 37% and median overall survival time 9.2 months. However, grade 3 or 4 treatment-related adverse events occurred in 69% of patients on DCF therapy [9,10].

Chemotherapeutic regimens including S-1 have recently produced good clinical responses and survival benefits in patients with gastric cancer in Japan. These positive responses were also observed in patients with non-resectable advanced-stage gastric adenocarcinoma [8]. The efficacy of chemotherapeutic regimens including S-1 has also been demonstrated in cases of gastric cancer [16]. S-1 has many advantages, including its high efficacy, excellent tolerability, low side-effect profile, and suitability for administration in an outpatient setting. Furthermore, its efficacy has been demonstrated in combination with CDDP for stage IV gastric adenocarcinoma [13] and in neoadjuvant chemotherapy with CDDP for unresectable advanced-stage gastric cancer [14,17]. To our knowledge, however, the effectiveness of chemotherapy with S-1 and CDDP against esophageal adenocarcinoma has not been previously studied. Given the positive response of other tumors, we decided to use this chemotherapy regimen in our patient with advanced-stage SDP esophageal and gastric adenocarcinoma. There has been a reported case of SDP esophageal squamous cell carcinoma and gastric adenocarcinoma in which a complete response was obtained for esophageal carcinoma to treatment with this regimen [18]. That case was different from this report as the carcinoma was at an early stage and was a squamous cell carcinoma. However, these results suggest that chemotherapy with S-1 plus CDDP may be effective in esophageal cancer.

Generally, the initial treatment for esophageal cancer is either chemotherapy or surgical resection, with or without esophageal preservation. However, esophagectomy results in dysphagia more commonly than does chemotherapy [19]. Furthermore, esophagectomy is associated with high mortality and morbidity. Even at high-volume centers, a 5% surgical mortality rate has been reported [20]. Therefore, chemotherapy with S-1 and CDDP may offer functional and prognostic merits over esophagectomy in patients with advanced-stage esophageal adenocarcinoma.

## Conclusion

This case confirms the potential for a complete response to S-1 and CDDP chemotherapy in patients with advanced-stage SDP esophageal and gastric adenocarcinoma. The accumulation of further such cases may enhance our understanding of this phenomenon and lead to the development of new treatment strategies for advanced-stage esophageal adenocarcinoma.

## Consent

Written informed consent was obtained from the patient for publication of this case report and accompanying images. A copy of the written consent is available for review by the Editor-in- Chief of this journal.

## Competing interests

The authors declare that they have no competing interests.

## Authors’ contributions

YM reviewed relevant literature and wrote the initial draft. MK wrote and reviewed the initial draft. MF provided clinical expertise and reviewed the manuscript. NK contributed the CT scans images. TK performed the chemotherapy and reviewed the manuscript. YM performed the chemotherapy and reviewed the manuscript. TF performed the chemotherapy and reviewed the manuscript. TM performed the chemotherapy and reviewed the manuscript. TT provided clinical expertise and reviewed the manuscript. All authors read and approved the final manuscript.
